# Role of TNF*-α* in the Pathogenesis of Migraine

**DOI:** 10.1155/2024/1377143

**Published:** 2024-01-03

**Authors:** Amrit Sudershan, Srishty Sudershan, Isha Sharma, Hardeep Kumar, Rakesh K. Panjaliya, Parvinder Kumar

**Affiliations:** ^1^Department of Human Genetics, Sri Pratap College Srinagar, Cluster University Srinagar, Srinagar 190001, Jammu and Kashmir, India; ^2^Institute of Human Genetics, University of Jammu, Jammu 180006, Jammu & Kashmir, India; ^3^Department of Zoology, University of Jammu, Jammu 180006, Jammu & Kashmir, India; ^4^Department of Neurology, Super Specialty Hospital, Jammu 180006, Jammu and Kashmir, India

## Abstract

**Background:**

Neurogenic neuroinflammation has a wide role in migraine pathogenesis including the transition from episodic migraine to chronic one. The seed molecule of neurogenic neuroinflammation, i.e., the TNF-*α* proinflammatory molecule, has gathered a lot of attention. This pleiotropic cytokine is a classical component of inflammatory soup, secreted by the microglial cell, and promotes a wide range of inflammatory reactions.

**Aim:**

In this review, we aimed to provide a culminating and comprehending glimpse into the TNF-*α* in association with the migraine.

**Method:**

A systematic literature survey method with a mixture of keywords was utilized to grasp the different elements that represent the association between TNF-*α* and migraine. *Discussion*. Highlighted the probable involvement of the TNF-*α* with migraine, the complexity of the matter such as activation of NF-KB signaling cascade, autoactivation, sensitization, and increased likelihood of transition cannot be neglected. Being TNF-*α* as a core node, it becomes the factor for linking diseases such as chronic inflammatory disorders, including COVID-19, and also interaction with other genes to develop severe conditions.

**Conclusion:**

To this end, TNF-*α* plays a critical role in chronification, and inhibiting its signaling would likely be a crucial strategy for migraine therapy.

## 1. Introduction

It is not strange to hear the term “migraine,” which has been around since Hippocrates' time. Since then, the definition has expanded dramatically and is now defined as “a brain condition featured with low neuronal hyperexcitability and vascular dysfunction as a consequence leading to the unwanted severe head pain (the cardinal feature)” [1]. Regarding its prevalence and disability, the Global Burden Disorder-2019 (GBD-2019) has enlightened that the general prevalence is about 24% (GBD 2019) with a variation related to the gender difference with the majority of females than males [2, 3]. This gender prevalence diversity is mainly due to hormonal differences. If we take disability as a concern, the GBD-2019 has provided significant data on it, presenting the highest disability-adjusted life years (DALYs) per 100,000 in Italy (775.25) preceded by Germany (729.25), Thailand (724.5), and with the lowest rate in Ethiopia (266), and in India on average of 552.79 with the highest in Sikkim (592.86) and the lowest in Bihar (513.9) (Homepage | The Institute for Health Metrics and Evaluation (healthdata.org).

Apart from such a high prevalence estimate, migraine is still a matter of debate regarding its pathological mechanism, development of diverse phenotypes, and progression toward chronification. Several pathological mechanisms have already been identified including cortical spreading depression (CSD), activation and sensitization of trigeminovascular foci, and activation of glial cells [4–7]. It is accepted that the hypersensitiveness of neurons, i.e., “lower threshold of neuronal hyperexcitability,” is the reason for the migraine attack initiation [8]. But still, the matter of fact that cannot be denied is that the seed pathological mechanisms that make migraine so much more complicated disorder and force the researcher to contemplate its cure which is still lacking.

When compared to the other 369 diseases and injuries in the world, headache is ranked as the third most disabling (Global Burden of Disease-2019). It is induced by the activation and sensitization of the trigeminovascular system, which is responsible for the headache phenotype and its sensitization, respectively [6]. CSD is responsible for the activation, but various molecules are involved in sensitizations which are collectively called inflammatory soup [7]. CSD is an abnormal intrinsic neurophysiological phenomenon with a dramatic shift in cortical steady potential [6] and is responsible for the aura. It is responsible for the loss of function of the cerebral cortex mainly due to the increased extracellular potassium, a neurotransmitter (glutamate), as well as inflammatory markers such as calcitonin gene-related peptide (CGRP), substance P (SP), bradykinin, histamine, prostaglandin, and TNF-*α* which are collectively categorized under inflammatory soup (IF) [9].

CSD is also responsible for secondary damage in the brain's vital structure including blood-brain barrier (BBB) damage [10] which enhances the sensitivity of neuronal populations near areas of contusive brain injury, as in models of localized cerebral ischemia and trauma [11]. Thus, brain immune cells play an important and fascinating part in keeping brain tissue in balance and fixing damage brought on by repeated CSD attacks and the abnormal buildup of “CSD consequences molecules.”

However, the subjects with a genetic predisposition in the genes of the inflammatory system might induce an abnormal inflammatory response, i.e., chronic inflammation (CI). CI has long been recognized as a significant cause of the disease [12]. Pieces of evidence have shown that TNF injection can cause headache, and TNF antibody has been shown to reduce pain in humans [13]. However, the purpose of attempting to write this review is to better understand migraine as a neuroinflammatory disorder referring to TNF-*α* as an example. Hence, we all know that inflammation cannot be covered in a single article. TNF-*α* is only the tip of the iceberg in the inflammatory response, and it cannot be overlooked that it still plays a significant role.

## 2. Material and Method

**Figure 1:**
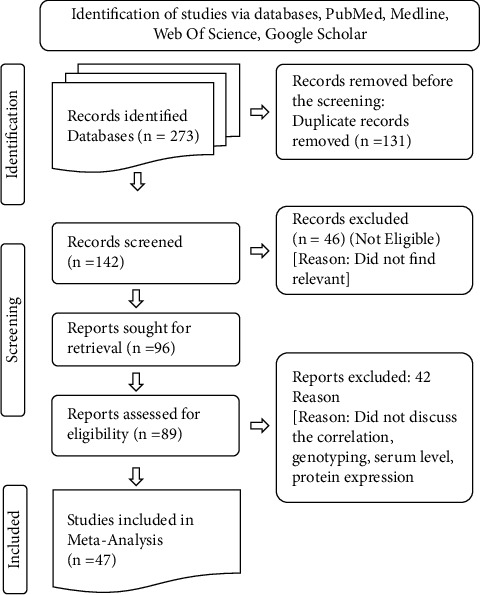
Systematic representation of literature survey according to PRISMA criteria.

For the literature search, we used the connected paper tool (https://www.connectedpapers.com/) initially using the following search words: TNF-*α* and migraine, which revealed the network of related research. Following the analysis of the article in the network, a selection was made based on TNF-*α* genotyping research, serum level research, protein expression, etc. We also used the standard approach of literature surfing for confirmation and increased accuracy, i.e., surfing in electronic databases such as Web of Science, Google Scholar, PubMed, Springer, and Elsevier until Feb. 2022. The relevant writers (AS, SS IS, HK, and PK) independently verified the data's validity. Keywords that were used include “migraine as a neurological disorder,” “polymorphism in TNF-*α* gene associated with migraine,” “TNF-*α*-308 promoter polymorphism associated with migraine,” “G-*308A* polymorphism of TNF-*α* in migraine,” “rs1800629 association with migraine,” “neuroinflammation role in migraine,” “microglial and migraine,” and TNFA expression in migraine model.” Only articles published in the English language were evaluated using the linguistic filters as a factor for publication selection. Primary and secondary research sources including scientific work, meta-analysis, and systematic reviews were critically reviewed. The study excluded incomplete data or only partially available. Because partial and missing data are not included in the study, there is no such detrimental effect, and we did our utmost to eliminate any undesirable characteristics. This narration includes a significant quantity of current material and follows PRISMA guidelines (PRISMA (prisma-statement.org) (Figure 1)).

## 3. Background

**Figure 2:**
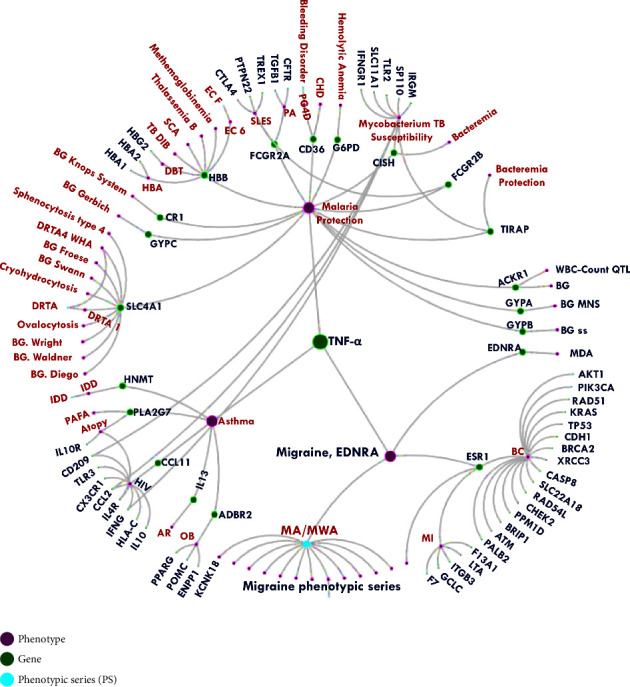
OMIM radial PheneGene graphs depicts the complex interaction of the TNF-*α* with different gene and are responsible for the diverse number of diseases and also with the migraine (highlighted with star) (OMIM-Online Mendelian Inheritance in Man (https://www.omim.org/)).

Chronic CNS (central nervous system) inflammation is characterized as a reaction that occurs as a result of deliberate injury, such as the breakdown of the BBB, and results in the activation of immune cells in the brain, such as microglia and astrocytes [14]. Such activation releases nitric oxide, glutamate, and proinflammatory cytokines [15]. When it comes to proinflammatory cytokines, TNF-*α* is one of the best examples. It starts inflammation and helps keep the body's balance. It is an undeniable truth that an excess of TNF-*α* may pose a major threat to the system, resulting in a variety of disorders (Figure 2). As a result of its core activity in inflammation [16], it is regarded as a hotspot molecule that has been investigated for decades.

TNF-*α* is secreted by the microglial cell upon activation by the release of histamine and serotonin, and changes in the level of TNF-*α* [7] across subjects can be a key risk factor for migraine susceptibility and severity. The researchers have been captivated by its role in neurogenic neuroinflammation [17]. Neurogenic neuroinflammation has a wide role in migraine pathogenesis which has been thought to have a critical role in the pathogenesis [18].

### 3.1. TNF-*α*

**Figure 3:**
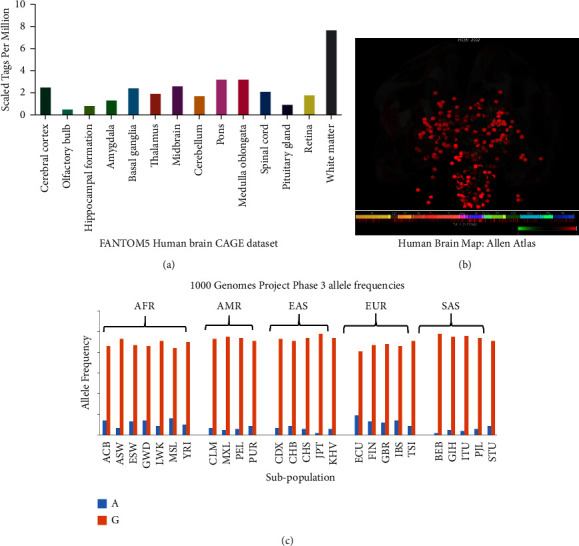
(a) Brain atlas has enlightened that TNF-*α* has a high expression in the various part of same including white matter, medulla oblongata, and pons (brain tissue expression of TNF-Summary-The Human Protein Atlas (https://www.proteinatlas.org/ENSG00000232810-TNF/brain)). (b) Microarray Data Allen Brain Atlas: human brain (brain-map.org. (c) −308G > A allele worldwide frequency in different population including African (AFR), American (AMR), EAS (East Asia), EUR (Europe), and SAS (South Asia) (Ensembl genome browser 105 (https://asia.ensembl.org/index.html)).

TNF-*α* (NCBI Entrez Gene: 7124) encodes for a pleiotropic cytokine that promotes a wide range of proinflammatory reactions and is responsible for various conditions including migraine (Figure 2) (MalaCards—human disease database; OMIM—Online Mendelian Inheritance in Man). Studying the expression of TNF-*α* from different expression databases (Brain tissue expression of TNF-Summary-The Human Protein Atlas), it is found that higher expression of the same is in the white matter (Figure 3(a)) which is characterized by the presence of myelinated nerve fibers. Using Allen Human Brain Atlas (Microarray Data—Allen Brain Atlas: human brain (brain-map.org), shows the increased expression of the TNF-*α* (probe: *A*_23_P376488) in different subcortical regions of the brain (Figure 3(b)) but shows a significantly higher expression in the medullary reticular formation (donor: H0351.2002: medullary reticular formation *Z*-score: 14.67, log2-level:12.71). The reticular formation is a net-like structure made up of various brainstem nuclei and neurons that cover a large portion of the brainstem. The network is distributed throughout the brainstem as an interconnected network of neurons with many projections rostrally to subcortical and cortical brain structures as well as caudally to the spinal cord. Interneurons in the lateral and medial tegmental fields, such as trigeminal, facial, vagal, and hypoglossal, have many functions, including the transmission of proprioceptive stimuli via descending axonal tracts [19].

At position −308 of the TNF-*α* promoter/enhancer region, a G to A transition polymorphism has been identified which shows different allele frequencies (rs1800629 (SNP): Explore this variant; Homo_sapiens: Ensembl genome browser 110). TNF1 or 308.1 nomenclatures are used to denote the more frequent 308G allele whereas the less common 308A allele is known as TNF2 or 308.2 and shows the allelic diversity within the different populations (Figure 3(c)). The rare 308A allele has been related to greater baseline/constitutive and inducible TNF-*α* expression both in vivo and in vitro [20] and is associated with elevated plasma levels [21].

For identifying the risk variants that cause the disease of interest, the “candidate gene studies approach” has been at the forefront in uncovering the correlation between genetic variants with complex diseases [22]. Multiple meta-analyses and other researchers have found a relationship between TNF-*α* 308GandA polymorphism with variations in susceptibility to several diseases between different people and, on a wider scale, across different populations [23–26].

### 3.2. TNF-*α* in Association with Migraine

**Figure 4:**
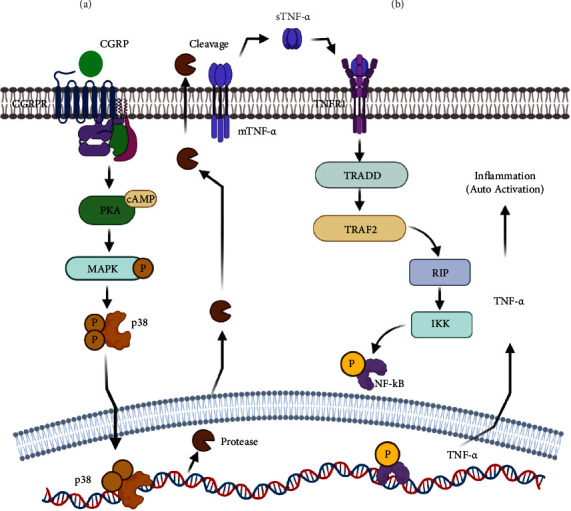
(a) The cycle begins with the binding of CGRP to its receptor, the CGRPR, which activates PKA (protein kinase A), which then phosphorylates the MAPK (mitogen activating protein kinase). When MAPK is phosphorylated, it causes p38 expression to increase as a result of the phosphorylation. The phosphorylated form of p38 subsequently enters the nucleus, where it stimulates the production of inflammatory genes such as TNF-*α*, protease. (b) After enhanced expression of protease, cleavage of mTNF-*α* is cleaved which after then binds with the TNFR1. Cascade of signal protein activation occurs after binding of sTNF-*α* to its TNFR1 which leads to the activation of IKK. IKK is responsible for the activation of NF-KB and activated form of protein enters into the nucleus and cause enhanced inflammation reaction.

Continuous pieces of evidence of an association between the TNF gene and migraine have been collected, with the most noteworthy finding coming from Franceschini and colleagues. They showed that LPS injection significantly increased TNF and macrophage production in Cav2.1R192Q (Familial Hemiplegic Migraine) KI (knock-in) ganglia when compared to WT (wild-type) ganglia. According to their findings, TNF-*α* may have a role in neuronal sensitization via altering crosstalk between sensory neurons and resident glial [27]. The addition of CGRP (Figure 4(a)) to primary trigeminal ganglion cells boosted the levels of many cytokines, including TNF-*α* [28, 29]. Concerning the expression analysis, Taheri and colleagues, after utilizing the expression assays for finding the expression level of cytokines in migraine sufferers compared to healthy controls, observed increased *TNF-α* expression inpatients than controls [30]. Similarly, Sarchielli and his team found a temporary increase in TNF-*α* levels in the blood from the internal jugular in migraine patients who did not have an aura in the two hours following the start of their migraine [31]. Also, Tang and group have shown that genetic deletion of *TNF-α* or intraspinal trigeminal nucleus caudalis (Sp5C) injection of TNF*α* receptor antagonist completely blocks pain prolongation [32].

The candidate gene study approach has been at the forefront of uncovering the correlation of genetic variants with complex diseases [22]. Using the same, multiple research studies have been conducted over the previous two decades; however, contradictory results have been discovered (Table 1), which might be related to demographic diversification, ethnic diversity, and varied sample size. But, in the recent polled analysis, a nonsignificant association was observed with overall migraine but critical increases in the Asian ethnic group [50]. We cannot ignore the findings from the genome-wide association study (GWAS), which identified several inflammatory genes such as TSPAN2, MEF2D, NLRP1, JAM3, and NOTCH4, but the TNF-*α* was not replicated [51]. This inconsistency is mainly due to several reasons and one such important is the study design difference between the candidate gene approach and GWAS [52].

Plasma level of the same was found to be significantly higher in the patients compared to controls [53–56] which is also supported by the recent meta-analysis [30, 57] with contradiction [58–60]. It has been also seen that TNF-a levels fluctuate more among children than in adults, and this difference might be due to a long medical history of migraine in adult patients and frequent intake of analgesic drugs or prophylactic treatment [61]. The CSF level of TNF alpha was found to be significantly elevated in chronic migraine patients [62], but with it, there is a contradiction [63].

The fact that a greater level of TNF-*α* showed a positive connection between TNF-*α* levels in CSF and serum and the extent of developing brain infarction cannot be overlooked [64]. This can be easily correlated to the cause of dementia including Parkinson's and Alzheimer's disease (AD) [65, 66], where the shreds of evidence from the model organism also support it [67]. The story does not end here, the interaction of TNF-*α* with other genes is the culprit for the various diseases (Figure 2).

### 3.3. Source and Mechanism of TNF-*α* Action in Migraine

Microglia, which has macrophage-like properties, is thought to be significant immune cells and make up about 5–10% of the parenchyma of the brain and spinal cord [68]. It continuously searches for any infection or alteration in the brain and thus aids in the regulation of brain hemostasis. Notably, when microglial cells are activated, they differentiate into “M1” and “M2” microglia types [69]. The “M1” cell type is found to be responsible for releasing cytokines like IL1, TNF-*α*, and NO, while the “M2” phenotype helps tissue repair and regeneration [70]. But long, excessive, and persistent stimulation of microglial cells can cause harmful inflammations that help neurodegenerative and neoplastic diseases develop [71–73]. It has also been found that microglial activation is the main cause of migraine pain and that this activation happens through cortical spreading depression [74, 75].

Concerning the production of TNF-*α*, which is known to cause inflammation and is known for its keen role in neuroinflammation [76, 77], it is made as a transmembrane protein (tmTNF-*α*) and then changed into soluble TNF-*α* (sTNF-*α*) by a matrix metalloprotease TNF-*α*-converting enzyme [78]. TNF-*α* exerts its effects via binding to the extracellular domain of the TNFR1 receptor, triggering receptor trimerization and thus initiating the cascade of events. After receptors are trimerized, the adaptor protein TRADD is brought in which then brings in other adaptor proteins such as receptor-interacting protein (RIP) and TNF receptor-associated factor 2 (TRAF2) [79]. TRAF2 is presented in a complex interaction with E3 ligase (cIAP1 and cIAP2) in the cytoplasm. Due to the activity of E3 ligase, it can tag RIP protein with ubiquitin molecule to create a binding site for another E3 ligase “linked ubiquitin chain assembly complex” (LUBAC) [80]. To facilitate the recruitment of a different kinase, “transforming growth factor-activated kinase-1” (TAK1), LUBAC further tags RIP [81].

TAK1 is bound to its docking site via an adaptor protein known as TAK1 binding protein-2 (TBP-2) and a kinase called Inhibitor of the kappa B kinase (IKK) complex. IKK is responsible for tagging IKB (an inhibitor of NF-kB) and promoting its proteasomal degradation [82, 83]. Phosphorylation of the NF-kB inhibitor (IKB) triggers nuclear translocation of NF-kB dimers, where it controls the production of proinflammatory cytokines such as TNF-*α* and IL-1*β* [84] (Figure 4(b)). Another finding shows that epigenetics, like miR-342 activating NF-kB/p65 by TNF-*α*, causes more TNF-*α* and IL-1*β* to be released in an autocrine way by encouraging the breakdown of BAG-1, which is a negative inhibitor of proinflammatory NF-kB in microglia [85].

### 3.4. Comorbidities with Migraine in Association with TNF-*α*

Comorbidity, or the occurrence of two or more chronic illnesses at the same time, is another major factor of concern that should be taken into account [86]. People who have more than one common disease at the same time are more likely to get it because they share risk factors which can be genetic or environmental.

Taking TNF-*α* as a reference point, various disorders have been discovered to be comorbid with migraine. It is not only that the amount of the same has risen, which can be linked to the illness; there's also evidence from other chronic conditions, such as up-regulation of TNF levels in the trigeminal nociceptive pathway, that gut microbiota dysbiosis contributes to the chronicity of migraine-like pain [32]. Patients with psoriasis [87, 88], inflammatory bowel diseases (IBD) [89, 90], and rheumatoid arthritis [91] have a higher risk of migraines.

There are several lines of shreds of evidence which directs that COVID-19 has been linked with migraine headaches [92–94]. Thus, this can't be neglected due to the enhanced rise of proinflammatory molecules including TNF-*α* [95]. The major consequence of the immune system to the infection is the release of proinflammatory molecules which are collectively called cytokines storm. The cytokine storm featured the presence of IL-1, IL-2, IL-6, IL-8, and TNF-*α* [95]. Enhanced production of TNF-*α* during COVID-19 infection causes the server activation of the trigeminovascular system [96].

## 4. Discussion

Migraine is a complex disorder and is still a matter of concern despite large treatment modalities. Discussing its various aspects including an abnormal spreading depression of neuronal and glial cells gave a glimpse into the imitation of the attack cycle, which further leads to the activation of the trigeminovascular system [97]. The activation of the same generates head pain, which has been extensively researched. The chronification, which is more than just “one cup of tea” might be the product of a slew of molecules and is something that must be understood. It has been shown that several genes are responsible for defining one's susceptibility to migraine conditions, and these genes also indicated that they are population-specific [98, 99].

**Figure 5:**
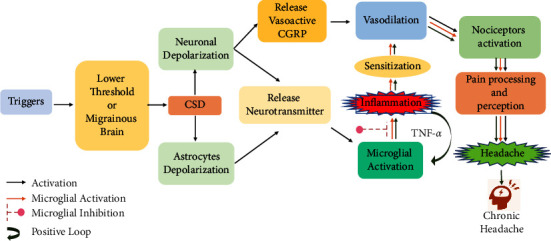
Flow diagram depicts how migrainous brain is triggered by slow propagating wave (2–5 mm·min^−1^) of all cortical neuronal and astrocyte depolarization leads to release of vasoactive product such as CGRP and neurotransmitter (glutamate). Microglial activation is caused by both CGRP and glutamate and stimulates the release of TNF-*α* which is the prime focus of inflammation. TNF-*α* also autostimulates and causes positive loop activation and is responsible for the sensitization of nociceptors, and this continuous stimulation will become a risk factor for the transition of episodic to chronic headache.

Repeated CSD increases the chance of secondary damage which hinders the sensitivity of neuronal populations [11]. Therefore, in maintaining the homeostasis of brain tissue and responding to injuries microglial cells play a significant role. However, genetic predisposition in the genes of the inflammatory system might be the cause for CI and TNF-*α* is the prime candidate which is seen to be higher in the subjects of migraine. Furthermore, the −308G > A polymorphism causes higher protein production and makes the individual more susceptible to persistent reactions (Table 1). Increased release of TNF-*α* by the microglial can enhance the activation of the entire population in a positive feed mechanism (Figure 5). Evidence has shown that increased TNF-*α* expression spontaneously develops chronic inflammatory demyelination with 100% penetrance [100]. This negative impact on demyelination and degeneration ultimately leads to neuronal damage. However, the irrefutable reality is that damaged neuronal populations release a substantial quantity of ATP, which binds to the P2X7 receptor on microglial cells and causes TNF-*α* to be produced and secreted in a de-novo manner [101]. There is additional evidence that TNF-*α* mediates activated microglial production of NO (nitric oxide), which inhibits neuronal respiration, resulting in glutamate release and consequent excitotoxicity [102]. TNF-*α*-induced glutamate cytotoxicity was achieved through two mechanisms: the autocrine microglial release and inhibition of the glutamate transporter on astrocytes.

Also, some data suggests that TNF-*α* raises the level of brain-derived neurotrophic factor (BDNF) in trigeminal ganglion neurons in a way that depends on their activity. These neurons are thought to be involved in how BDNF affects the plasticity of neurons [103]. Migraine sufferers have been found to have significantly higher amounts of BDNF compared to the healthy population, linking this protein not just to brain plasticity but also to the regulation and central sensitization of pain [104]. The BDNF variant rs2049046, located on the 5′ end of the same transcript, has been shown to have a strong correlation with migraine [105].

Therefore, the higher level of TNF-*α* and binding to TNFR1 initiate a series of downstream signaling proteins and thus transduce the signal. Furthermore, activation leads to the activation of kinases such as TAK1 and the IKK complex. IKK is responsible for the phosphorylation of IkB*α* (an inhibitor of NF-kB) and promotes its proteasomal degradation [82]. NF-*κ*B dimmers are the transcription factor which is the prime signaling protein that is being activated by the signaling pathway. Activated NF-B dimers are then translocated to the nucleus, where they regulate the production of proinflammatory cytokines such as tumor necrosis factor-alpha and interleukin-1 [84]. Autocrine signaling maintains the level of TNF-*α*, but this chronic level further decreases the threshold and increases the sensitivity.

To this end, polymorphism in the TNF-*α* would result in increased protein production, and persistent inflammation would most likely be caused by TNF-positive loop activation. TNF-*α* just one molecule among many and represents just the “tip of the iceberg” but it still presents a significant role in neurogenic neuroinflammation. Accordingly, neurogenic neuroinflammation is becoming acknowledged as a potential pathogenic process that may have the potential to control the severity of the disease and categorize the condition as an inflammatory disorder.

## 5. Future Perspective

Praise to the research that has revealed various treatment strategies, but still all relate to the symptomatic treatment. Various new treatment strategies have been now discovered which probably increases the chance of treatment. Anti-TNF therapy has been used for treating various diseases for a long period [66, 106–108]. The usage of Venlafaxine in headaches, which was dosage- and time-dependent, resulted in pain relief [109]. Furthermore, Abdolahi and his colleagues reported that taking extra *β*-3 fatty acids and curcumin could be a new, promising way to treat migraines because the two together greatly decreased TNF-*α* messenger RNA (mRNA) in a way that worked well with each other [110]. As a result, there is a window of opportunity for developing novel migraine treatment options related to the TNF-*α* signaling pathway. The significant involvement in maintaining the autocrine loop mediated by the degradation of BAG-1 and miR-342 might be a possibility for a therapeutic approach in the TNF-*α* signaling pathway. This may aid in preventing the progression of episodic migraine to chronic migraine.

Enclosing the section, continued research for the discovery of genetic, epigenetic, and molecular biomarkers, is needed which help for identifying the diseases and also motivate personalized medicine to treat migraine.

## 6. Limitation

There is an urgent need for a meta-analysis for the −308G > A, which would likely enhance statistical power and shed light on the strong association between the variant and migraine. Also, the facts remain unanswered, including whether indiscriminate use of anti-TNF medicines might alter the disease's natural course and whether anti-TNF therapy should be used alone or in conjunction with immunomodulators over time. Although biomarker research and validation have significantly improved diagnostic precision and measures of therapeutic efficacy, there are currently no biomarkers for chronic or episodic migraine.

## 7. Conclusion

For a long period, TNF-*α* is the primary candidate when linking neurogenic neuroinflammation and migraine. Activation of the microglial and auto-activation loops of TNF-*α* significantly influences the chronicity and disease severity. As a result, it is obvious that neurogenic neuroinflammation plays a substantial role in migraine pathogenesis and that microglial activation plays a vital role in disease development in terms of TNF-*α* signaling.

## Figures and Tables

**Table 1:** Features of different association study utilizing case-control and cohort study design.

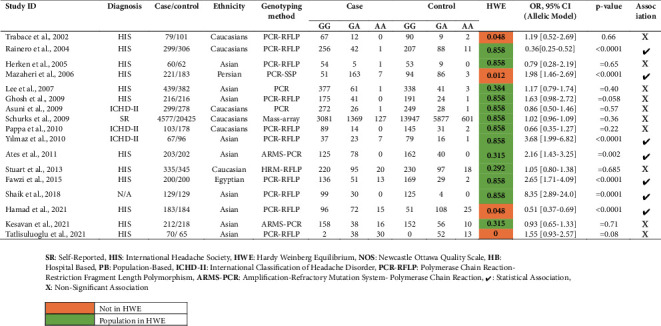

## Data Availability

No data were used to support this study.

## Abbreviations

DALYs: Disability-adjusted life years
GBD: Global burden disorder
CSD: Cortical spreading depression
WHO: World Health Organization
CGRP: Calcitonin gene-related peptide
SP: Substance P
TNF-*α*: Tumor necrosis factor alpha
IF: Inflammatory soup
BBB: Blood-brain barrier
CNS: Central nervous system
CI: Chronic inflammation
KI: Knock-in
WT: Wild type
TSPAN2: Tetraspanin 2
MEF2D: Myocyte enhancer factor 2D
NLRP1: NLR family pyrin domain containing 1
JAM3: Junctional adhesion molecule 3
NOTCH4: Notch receptor 4
TNFR1/2: Tumor necrosis factor receptor ½.
